# Pan-Enterovirus Amplicon-Based High-Throughput Sequencing Detects the Complete Capsid of a EVA71 Genotype C1 Variant via Wastewater-Based Epidemiology in Arizona

**DOI:** 10.3390/v13010074

**Published:** 2021-01-07

**Authors:** Temitope O. C. Faleye, Erin Driver, Devin Bowes, Sangeet Adhikari, Deborah Adams, Arvind Varsani, Rolf U. Halden, Matthew Scotch

**Affiliations:** 1 Biodesign Center for Environmental Health Engineering, Biodesign Institute, Arizona State University, Tempe, AZ 85287, USA; tfaleye@asu.edu (T.O.C.F.); Erin.Driver@asu.edu (E.D.); dbowes@asu.edu (D.B.); sadhik14@asu.edu (S.A.); rhalden@asu.edu (R.U.H.); 2 Biodesign Center for Personalized Diagnostics, Biodesign Institute, Arizona State University, Tempe, AZ 85287, USA; dadams14@asu.edu; 3 Biodesign Center for Fundamental and Applied Microbiomics, Biodesign Institute, Arizona State University, Tempe, AZ 85287, USA; Arvind.Varsani@asu.edu; 4 OneWaterOneHealth, Nonprofit Project of the Arizona State University Foundation, Tempe, AZ 85287, USA

**Keywords:** Enterovirus A, human, poliomyelitis, wastewater, molecular epidemiology, Arizona

## Abstract

We describe the complete capsid of a genotype C1-like Enterovirus A71 variant recovered from wastewater in a neighborhood in the greater Tempe, Arizona area (Southwest United States) in May 2020 using a pan-enterovirus amplicon-based high-throughput sequencing strategy. The variant seems to have been circulating for over two years, but its sequence has not been documented in that period. As the SARS-CoV-2 pandemic has resulted in changes in health-seeking behavior and overwhelmed pathogen diagnostics, our findings highlight the importance of wastewater-based epidemiology (WBE ) as an early warning system for virus surveillance.

## 1. Introduction

Enterovirus A71 (EVA71) is a member of Enterovirus A species in the genus *Enterovirus*, family *Picornaviridae*, order *Picornavirales*. Like other EVs, the EVA71 genome is a plus-sense, single-stranded, monopartite RNA with a length of ~7500 nts. Until recently, EV genomes were known to have one polyprotein (>2000 aa) encoding open reading frame (ORF) whose protein product is proteolytically cleaved first into three protein precursors (P1, P2 and P3) and subsequently into 11 proteins; VP1– VP4 (the capsid proteins from P1), 2A–2C (from P2) and 3A–3D (from P3). However, it has been shown that EV genomes also often encode a small ORF upstream of and overlapping the polyprotein ORF (ppORF) [1]. The upstream ORF (uORF) encodes a 56–76 aa peptide (UP) that has been shown to modulate gut infection [1]. Based on sequence diversity of the VP1 protein, seven (7) EVA71 genotypes (A–G) and several sub-genotypes (e.g., C1–C5) have been delineated [2].

EVA71 has been associated with hand, foot and mouth disease (HFMD) globally for over two decades [3] and more recently (alongside EVD68) with a polio-like illness called Acute Flaccid Myelitis (AFM). Since 2012, AFM epidemiology has shown biennial (2012, 2014, 2016 and 2018) peaks [3] and there have been over 600 confirmed cases in the USA with fourteen (1, 6, 2 and 5 in 2014, 2016, 2017 and 2018, respectively) of them in Arizona [4]. Despite the occurrence of AFM cases, there has been limited EVD68 and EVA71 genomic sequence data from Arizona in GenBank. The authors are only aware of one partial EVA71 VP1 sequence from Arizona recovered in 1994 (AF009553) [5]. Here, we describe the complete capsid of a genotype C1-like EVA71 variant from wastewater in a neighborhood in the greater Tempe, Arizona area (Southwest United States) on the 6th of May 2020.

## 2. Results

We identified a partial genome of EV-A71 genotype C1, GenBank accession MT952340, from 5.29% (169,428/3,205,226) of the reads, with a mean coverage of 7968X. The sequence (3956 nt) spanned the 5${}^{\prime }$-UTR to 2C genomic region (nucleotide position 538 to 4446 relative to NC_001612 reference sequence). A BLASTn [6] search of the GenBank [7] database using the complete contig, P1 and P2 showed the variant to be most similar to KU641507 (97.44%), KU641501 (97.60%) and LR027532 (97.30%), respectively. All (KU641507, KU641501 and LR027532) are EVA71 genotype C1 isolates recovered in Europe between 2015 and 2016 and documented to belong to a lineage that circulated in Europe from 2015 to 2019 causing HFMD with severe neurologic complications [8,9,10].

**Figure 1:**
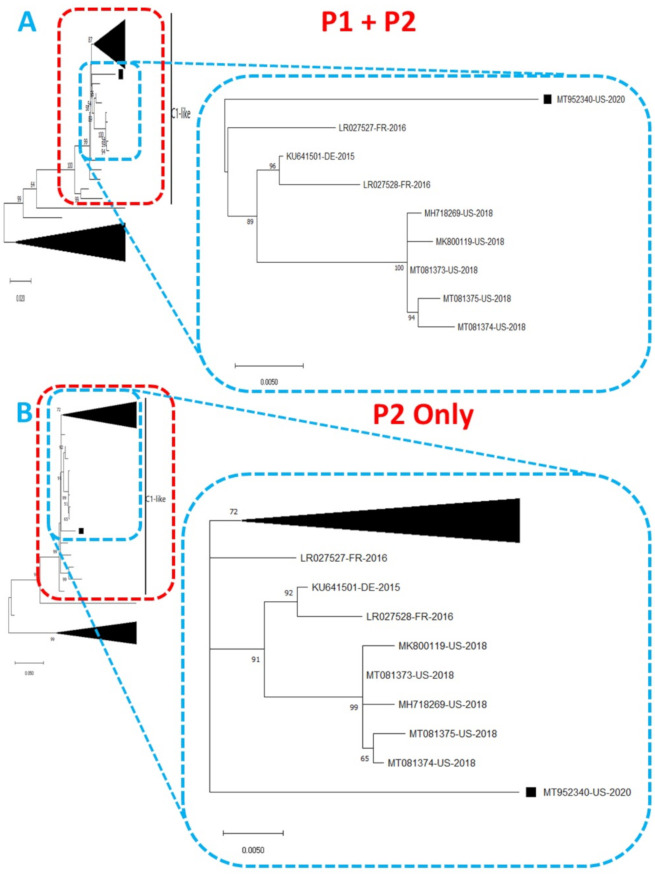
Phylogenetic tree of genetic relationship between (**A**) P1 + P2 and (**B**) P2 only nucleotide sequences of EV-A71 genotype C1. The phylogenetic trees are based on an alignment of nucleotide sequences of the respective genomic regions. The newly sequenced variant (MT952340, detected in wastewater in Arizona 2020) is indicated with a black square. Bootstrap values are indicated if >50%.

**Figure 2:**
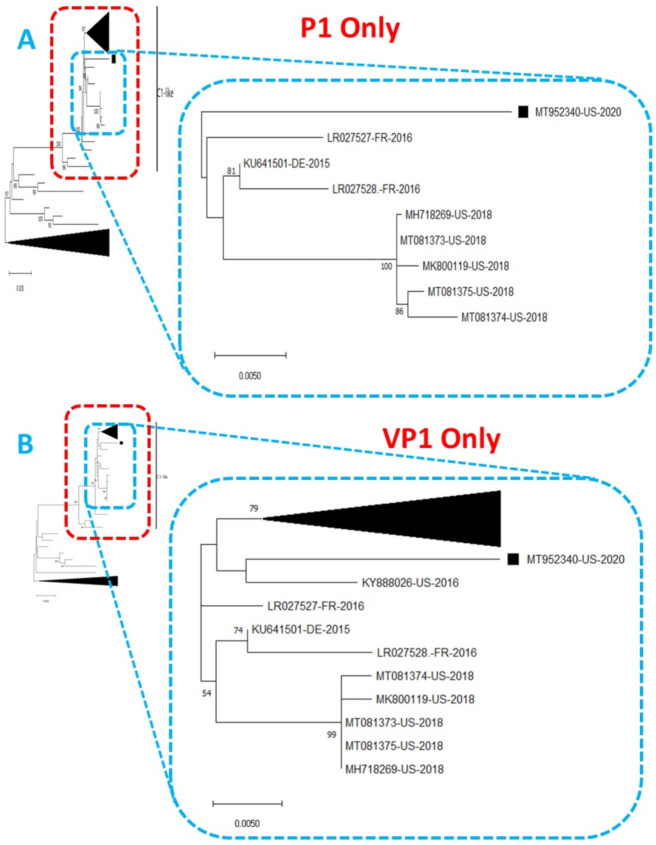
Phylogenetic tree of genetic relationship between (**A**) P1 only and (**B**) VP1 only nucleotide sequences of EV-A71 genotype C1. The phylogenetic trees are based on an alignment of nucleotide sequences of the respective genomic regions. The newly sequenced virus (MT952340, detected in wastewater in Arizona 2020) is indicated with a black square. Bootstrap values are indicated if >50%.

**Figure 3:**
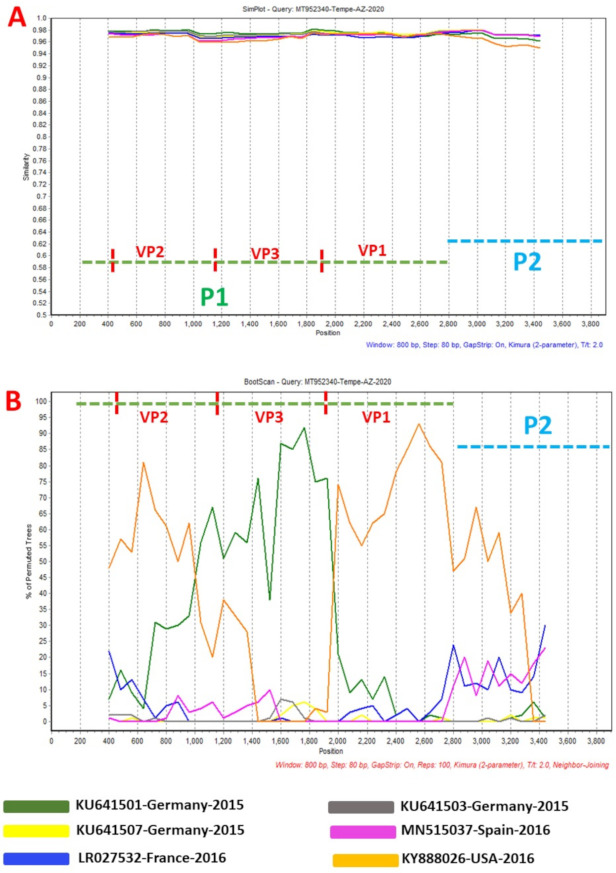
Similarity plot (**A**) and bootscanning analysis (**B**) using EVA71 C1-like sequences from GenBank. The green and blue dashed lines denote P1 and P2 genomic regions, respectively. MT952340 was used as query sequence. This analysis was done using the Kimura (2-parameter) model in SimPlot version 3.5.1 a sliding window of 200 base pairs moving in steps of 20 nucleotides.

Phylogentic analysis of the P1 and P2 regions from MT952340 suggests that it shares a common ancestor with the lineage that contains C1-like variants associated with severe neurological complications in Europe (from 2015–2016) and America (2018) (Figure 1 and Figure 2A). However, we found contrary results when examining VP1. Phylogenetic analysis using the complete gene suggests MT952340 is most closely related to a 2016 variant (KY888026) recovered in the USA (Figure 2B). This could be due to selection bias since most of the eighty (80) sequences analyzed here have a complete genome (or at least complete P1) but most sequences in GenBank have only VP1. Specifically, we searched GenBank using the query “Enterovirus A71 complete genome” or “Enterovirus A71 VP1” and found 1001 and 10,622 hits, respectively. To rule out this bias, we used the complete VP1 sequence of our variant (MT952340) in a BLASTn search of GenBank and selected the top 100 hits. We removed 39 sequences that were already in our dataset and used the 61 remaining, plus our dataset of 80 sequences, to construct a new ML tree. The result (Figure S1) shows a similar topology as Figure 2B with MT952340 clustering with the 2016 variant (KY888026) recovered in the USA. These results, taken together, suggest that MT952340 has recombination occurring within the P1 genomic region. To further examine this hypothesis, we performed similarity and bootscan analysis in Simplot [11]. Simplot showed that MT952340 was indeed a C1-like EVA71 both in the P1 and P2 genomic regions (Figure 3A). Bootscan analysis however suggest that MT952340 might have a complex recombination history even within the C1-like sub-genogroup (Figure 3B). Specifically, it suggested that P1 was (at least) a double recombinant. We tested this hypothesis by making ML trees of VP1, VP2 and VP3 and these confirmed the results of bootscanning by showing different topologies per gene (Figures S2–S4). In addition, pairwise distance analysis using complete VP1 sequences showed that all the EVA71 variants in GenBank were >2% divergent from MT952340 (data not shown). In poliovirus surveillance parlance, this would have been classified an ‘orphan virus’ [12], suggesting gaps in case-based surveillance. Going by the molecular clock of EVs [13,14], the fact that all the EVA71 variants in GenBank were >2% divergent from MT952340 suggests that a predecessor variant circulated and consequently accumulated mutations for over two years to result in the sequence MT952340 (the variant described here). More importantly, none of the variant sequences spanning the over two-year evolution from the predecessor variant to MT952340 have been made publicly available, if sequenced. Hence, as of the time of writing, analysis of MT952340 using publicly available sequence data in GenBank shows that there is a two-year gap of undescribed circulation of EVA71 C1-like variants related to MT952340. Whether these strains have been detected by different research groups, but are yet to be deposited in public databases such as GenBank, remains to be determined. This could be long enough to accumulate the complex recombination history suggested by the results of Bootscan and phylogenetic analysis (Figure 1, Figure 2 and Figure 3, Figures S1–S4).

This recombination pattern could have been as a result of template switching during PCR amplification. This would imply the presence of more than one variant in our sample. However, we found no single nucleotide variants or INDELS in the reads (data not shown). Furthermore, we Sanger-sequenced a 350 bp portion of VP1, and viewed the chromatogram which had clean peaks and the same sequence as MT952340 (Figure S5). This further confirmed our virus consists of one variant and not an assemblage of variants. Interestingly, the only difference at the VP1 amino acid (aa) level between MT952340 and virus sequences from the European and United States is an asparagine to serine substitution at position 282 (N282S) in the VP1 protein (Figure S6). It is not clear how this change might impact the phenotype of the virus but changes (usually N282D) in this VP1 aa position has been associated with increased virus replication in both human (rhabdomyosarcoma) and nonhuman primate (kidney; Vero) cell lines [15,16]. Also, there are six (6) substitutions (V28I, Y35H, N59S, K65R, L70P and V73A) in the UP that seem unique to MT952340 (data not shown). Considering UP’s relatively new discovery, it is not clear how these amino acid substitutions in the UP protein might impact its function.

## 3. Discussion

We describe an EVA71 C1-like variant (MT952340) detected in wastewater in a neighborhood in the greater Tempe, Arizona area (Southwest United States) in May 2020. MT952340 has a complex recombination pattern that suggests it has regions from variants in Europe and USA and seems to have been circulating for over two (2) years undetected. Recently, Ngagas et al. [10] showed that four (4) recombination modules (5${}^{\prime }$-UTR, P1, P2 and P3) might exist in the EVA71 C1-like genome. Our data (Figure 3B) suggests the number of recombination modules might be more. Specifically, while the four (4) modules detailed in Ngagas et al. [10] might be true for inter-genotype recombination, our data suggests that intra-subgenotypically, the P1 module in EVA71 C1-like genomes might function as at least three modules as has been previously shown for P1 segments of EV-A, EV-B and EV-C [17,18,19]. Altogether, the results of this study suggest several things. First is that more EVA71 C1-like variants exist than is currently documented by case-based surveillance (CBS). This is not surprising as CBS usually only captures only 0.5–1% of people infected with EVs [20]. Secondly, our data suggests that coinfection with multiple variants of EVA71 C1-like happens as this seems the only way the type of recombinant described here could have evolved. Thirdly, as has been previously documented for EV-A, EV-B and EV-C [17,18,19], it seems the P1 genomic region of EVA71 C1-like genomes also function as multiple recombination modules within the subgenotype.

Despite the SARS-CoV-2 lockdown, the CDC has confirmed 29 cases of AFM across the USA in 2020 [4] (none in Arizona); suggesting there was EV activity during this time. Considering the biennial peak pattern of AFM, 2020 was supposed to be an AFM peak-year. It is, therefore, possible that the lockdown of schools and daycare centers, which are the major transmission hubs of EVs, and the consequent reduced host mobility intended to curb the spread of SARS-CoV-2, might have unexpectedly also helped to do the same for EVA71. It is also likely that the overwhelming burden of SARS-CoV-2 in the United States has resulted in changes in health-seeking behavior (see [21]) and testing bias towards COVID-19 as opposed to other possible infections. It is, therefore, possible that there had been silent circulation of EVA71 C1-like in the greater Tempe, Arizona area in 2020 which did not show up on the case-based surveillance radar.

Our findings highlight how valuable WBE can be for enhancing our ability to catalogue EVA71 C1-like diversity and consequently, our understanding of its evolutionary dynamics. Furthermore, it also shows the importance of wastewater-based epidemiology as an early-warning system for detecting the introduction or circulation of viruses within communities.

## 4. Materials and Methods

We analyzed wastewater as part of routine wastewater-based epidemiology (WBE) via the Human Health Observatory (HHO) [22], Biodesign Institute, Arizona State University (ASU). The HHO represents a network of greater than 300 cities across the globe [23] that routinely provides wastewater and sludge samples for analysis and research. The specific sample described here was a composite sample collected using a time-weighted automated sampler which aliquots approximately 60 mL of wastewater every 15 min over a 24-h period. The wastewater collection station sampled in this study of a neighborhood in Arizona in the greater Tempe area serves the total population of 6500 residents. The sample was then transported on ice to the wastewater processing laboratory at the Biodesign Institute, ASU where 400 mL of the sample was subjected to virus concentration using polyethylene glycol (PEG) 8000 and sodium chloride precipitation. Subsequently, RNA was extracted from the concentrate and subjected to the one-step reverse-transcriptase polymerase chain reaction (RT-PCR) assay recently described [24] using the SSIII, one-step RT-PCR kit with Platinum Taq (Invitrogen, Carlsbad, CA, USA). Part of the amplicon was used as template in a nested PCR assay using primers AN89 and AN88 as detailed in Nix et al. [25]. Both amplicons were submitted to the ASU Genomics Core. The first round PCR amplicon (~4 kb) was used for library preparation (KAPA Hyperplus Library Kit) followed by paired end sequencing (2 × 250 bp) using the MiSeq system V2 (Illumina, San Diego, CA, USA). The nested PCR amplicon (~350 bp) was Sanger-sequenced using primers AN89 and AN88.

The Illumina raw reads were viewed using FASTQC v0.11.5, trimmed using Trimmomatic v0.36 and de novo assembled using MetaSpades v3.13.0 (all using default parameters) on the KBase platform [26]. The EV contig in the assembly was identified using the enterovirus genotyping tool [27] and then used for variant analysis in Geneious Prime (using the built-in “Find Variations/SNPs” program) [28].

For phylogenetic analysis, the EV contig recovered in this study was used for a BLASTn search of the GenBank database. The top 100 hits were downloaded from GenBank and further screened using the enterovirus genotyping tool [27] to remove those that did not belong to the same subgenotype. Subsequently, we had eighty (80) sequences (including ours) in a local database (see Table S1 for a list of all sequences analyzed in this study). These 80 sequences were subjected to multiple sequence alignment using the ClustalW program in MEGA X software [29]. Subsequently, maximum-likelihood (ML) trees were constructed using 1000 bootstrap replicates in MEGA X [29] for the P1 + P2, P2, P1, VP2, VP3 and VP1 genomic regions. The pairwise distance calculator in MEGA X [29] was used for VP1 pairwise distance estimation. This was done using the Kimura (2-parameter) model and 1000 bootstrap replicates. The complete EV contig was also subjected to similarity and bootscanning analysis using the Kimura (2-parameter) model in SimPlot version 3.5.1 [11] with a sliding window of 800 base pairs moving in steps of 80 nucleotides.

The sequence described in this study has been submitted in GenBank under the accession number MT952340. The raw reads have been deposited under SRA accession number SRX9713168.

## Data Availability

The data presented in this study are openly available in SRA under accession number SRX9713168 and in GenBank under accession number MT952340. In Table S1, we provide GenBank accession numbers of the sequences analyzed in this study.

## Supplementary Materials

The following are available online at https://www.mdpi.com/1999-4915/13/1/74/s1, Figure S1: Phylogenetic tree of EVA71 genotype C1; Figure S2: Phylogenetic tree on an alignment of VP1 nucleotide sequences of the 80 EVA71 genotype C1 sequences detailed in Table S1; Figure S3: Phylogenetic tree based on an alignment of VP3 nucleotide sequences of the 80 EVA71 genotype C1 sequences detailed in Table S1; Figure S4: Phylogenetic tree based on an alignment of VP2 nucleotide sequences of the 80 EVA71 genotype C1 sequences detailed in Table S1; Figure S5: Chromatogram of Sanger sequencing results of a 350 bp amplicon of the VP1 gene for MT952340; Figure S6: Amino acid translation of 11 complete VP1 sequences (297 aa) from select isolates in Europe and the United States; Table S1: Sequences analyzed in this study.

## Abbreviations

The following abbreviations are used in this manuscript:

AFM	acute flaccid myelitis
ASU	Arizona State University
COVID-19	Coronavirus Disease 2019
EGT	enterovirus genotyping tool
EV	enteroviruses
EVA71	enterovirus A71
HFMD	hand, foot, and mouth disease
HHO	Human Health Observatory
HTS	high-throughput sequencing
ML	maximum-likelihood
MSA	multiple sequence alignment
ORF	open reading frame
PEG	polyethylene glycol
ppORF	polyprotein open reading frame
RT-PCR	reverse transcription polymerase chain reaction
SARS-CoV-2	severe acute respiratory syndrome coronavirus 2
uORF	upstream open reading frame
USA	United States of America
WBE	wastewater-based epidemiology

## References

[1] Lulla V., Dinan A.M., Hosmillo M., Chaudhry Y., Sherry L., Irigoyen N., Nayak K.M., Stonehouse N.J., Zilbauer M., Goodfellow I. (2019). An upstream protein-coding region in enteroviruses modulates virus infection in gut epithelial cells. Nat. Microbiol..

[2] Bessaud M., Razafindratsimandresy R., Nougairede A., Joffret M.L., Deshpande J.M., Dubot-Peres A., Heraud J.M., de Lamballerie X., Delpeyroux F., Bailly J.L. (2014). Molecular comparison and evolutionary analyses of VP1 nucleotide sequences of new African human enterovirus 71 isolates reveal a wide genetic diversity. PLoS ONE.

[3] Uprety P., Graf E.H. (2020). Enterovirus infection and acute flaccid myelitis. Curr. Opin. Virol..

[4] CDC (2021). AFM Cases and Outbreaks. https://www.cdc.gov/acute-flaccid-myelitis/cases-in-us.html.

[5] Brown B.A., Oberste M.S., Alexander J.P.J., Kennett M.L., Pallansch M.A. (1999). Molecular epidemiology and evolution of enterovirus 71 strains isolated from 1970 to 1998. J. Virol..

[6] Altschul S.F., Gish W., Miller W., Myers E.W., Lipman D.J. (1990). Basic local alignment search tool. J. Mol. Biol..

[7] Sayers E.W., Cavanaugh M., Clark K., Ostell J., Pruitt K.D., Karsch-Mizrachi I. (2020). GenBank. Nucleic Acids Res..

[8] Antona D., Kossorotoff M., Schuffenecker I., Mirand A., Leruez-Ville M., Bassi C., Aubart M., Moulin F., Levy-Bruhl D., Henquell C. (2016). Severe paediatric conditions linked with EV-A71 and EV-D68, France, May to October 2016. Eurosurveillance.

[9] Böttcher S., Obermeier P.E., Neubauer K., Diedrich S. (2016). Laboratory Network for Enterovirus Diagnostics. Recombinant Enterovirus A71 Subgenogroup C1 Strains, Germany, 2015. Emerg. Infect. Dis..

[10] Ngangas S.T., Lukashev A., Jugie G., Ivanova O., Mansuy J.M., Mengelle C., Izopet J., L’Honneur A.S., Rozenberg F., Leyssene D. (2019). Multirecombinant Enterovirus A71 Subgenogroup C1 Isolates Associated with Neurologic Disease, France, 2016–2017. Emerg. Infect. Dis..

[11] Lole K.S., Bollinger R.C., Paranjape R.S., Gadkari D., Kulkarni S.S., Novak N.G., Ingersoll R., Sheppard H.W., Ray S.C. (1999). Full-length human immunodeficiency virus type 1 genomes from subtype C-infected seroconverters in India, with evidence of intersubtype recombination. J. Virol..

[12] Snider C.J., Diop O.M., Burns C.C., Tangermann R.H., Wassilak S.G. (2016). Surveillance Systems to Track Progress Toward Polio Eradication–Worldwide, 2014–2015. MMWR Morb. Mortal. Wkly. Rep..

[13] Jorba J., Campagnoli R., De L., Kew O. (2008). Calibration of multiple poliovirus molecular clocks covering an extended evolutionary range. J. Virol..

[14] Fernandez-Garcia M.D., Kebe O., Fall A.D., Dia H., Diop O.M., Delpeyroux F., Ndiaye K. (2016). Enterovirus A71 Genogroups C and E in Children with Acute Flaccid Paralysis, West Africa. Emerg. Infect. Dis..

[15] Caine E.A., Moncla L.H., Ronderos M.D., Friedrich T.C., Osorio J.E. (2016). A Single Mutation in the VP1 of Enterovirus 71 Is Responsible for Increased Virulence and Neurotropism in Adult Interferon-Deficient Mice. J. Virol..

[16] Mandary M.B., Masomian M., Ong S.K., Poh C.L. (2020). Characterization of Plaque Variants and the Involvement of Quasi-Species in a Population of EV-A71. Viruses.

[17] Lukashev A.N., Lashkevich V.A., Ivanova O.E., Koroleva G.A., Hinkkanen A.E., Ilonen J. (2005). Recombination in circulating Human enterovirus B: Independent evolution of structural and non-structural genome regions. J. Gen. Virol..

[18] Huang S.W., Hsu Y.W., Smith D.J., Kiang D., Tsai H.P., Lin K.H., Wang S.M., Liu C.C., Su I.J., Wang J.R. (2009). Reemergence of enterovirus 71 in 2008 in taiwan: dynamics of genetic and antigenic evolution from 1998 to 2008. J. Clin. Microbiol..

[19] Bessaud M., Joffret M.L., Holmblat B., Razafindratsimandresy R., Delpeyroux F. (2011). Genetic relationship between cocirculating Human enteroviruses species C. PLoS ONE.

[20] Nathanson N., Kew O.M. (2010). From emergence to eradication: The epidemiology of poliomyelitis deconstructed. Am. J. Epidemiol..

[21] Lange S.J., Ritchey M.D., Goodman A.B., Dias T., Twentyman E., Fuld J., Schieve L.A., Imperatore G., Benoit S.R., Kite-Powell A. (2020). Potential Indirect Effects of the COVID-19 Pandemic on Use of Emergency Departments for Acute Life-Threatening Conditions—United States, January-May 2020. MMWR Morb. Mortal. Wkly. Rep..

[22] Venkatesan A.K., Done H.Y., Halden R.U. (2015). United States National Sewage Sludge Repository at Arizona State University—A new resource and research tool for environmental scientists, engineers, and epidemiologists. Environ. Sci. Pollut. Res. Int..

[23] Halden R.U., Terlinden E., Kraberger S., Scotch M., Steele J., Varsani A. (2019). Tracking harmful chemicals and pathogens using the Human Health Observatory at ASU. Online J. Public Health Inform..

[24] Majumdar M., Martin J. (2018). Detection by Direct Next Generation Sequencing Analysis of Emerging Enterovirus D68 and C109 Strains in an Environmental Sample From Scotland. Front. Microbiol..

[25] Nix W.A., Oberste M.S., Pallansch M.A. (2006). Sensitive, seminested PCR amplification of VP1 sequences for direct identification of all enterovirus serotypes from original clinical specimens. J. Clin. Microbiol..

[26] Arkin A.P., Cottingham R.W., Henry C.S., Harris N.L., Stevens R.L., Maslov S., Dehal P., Ware D., Perez F., Canon S. (2018). KBase: The United States Department of Energy Systems Biology Knowledgebase. Nat. Biotechnol..

[27] Kroneman A., Vennema H., Deforche K.V.D., Avoort H.V.D., Penaranda S., Oberste M.S., Vinje J., Koopmans M. (2011). An automated genotyping tool for enteroviruses and noroviruses. J. Clin. Virol..

[28] Kearse M., Moir R., Wilson A., Stones-Havas S., Cheung M., Sturrock S., Buxton S., Cooper A., Markowitz S., Duran C. (2012). Geneious Basic: An integrated and extendable desktop software platform for the organization and analysis of sequence data. Bioinformatics.

[29] Kumar S., Stecher G., Li M., Knyaz C., Tamura K. (2018). MEGA X: Molecular Evolutionary Genetics Analysis across Computing Platforms. Mol. Biol. Evol..

