# Supplementary material for: Pan-Enterovirus Amplicon-Based High-Throughput Sequencing Detects the Complete Capsid of a EVA71 Genotype C1 Variant via Wastewater-Based Epidemiology in Arizona
Source: Viruses. 2021 Jan 7;13(1):74. doi: 10.3390/v13010074 (PMC7827028; doi:10.3390/v13010074)
Supplement: Supplementary file 1 [file viruses-13-00074-s001.zip › tableS1.pdf]

S/N	Name	begin	length	end	type	VP1 subgenotype	VP1 subgenotype support	Country	Year of Detection	Sample collected: Host
1	MT952340	538	3905	4446	EV-A71	C1	100	USA	2020	Wastewater: Not Applicable
2	KU641507	184	7157	7344	EV-A71	C1	100	Germany	2015	Stool: Human
3	KU641503	184	7156	7343	EV-A71	C1	100	Germany	2015	Stool: Human
4	KU641504	184	7157	7344	EV-A71	C1	100	Germany	2015	Stool: Human
5	KU641501	184	7152	7339	EV-A71	C1	100	Germany	2015	Stool: Human
6	KU641502	184	7160	7347	EV-A71	C1	100	Germany	2015	Stool: Human
7	KU641505	184	7156	7343	EV-A71	C1	100	Germany	2015	Stool: Human
8	KX139462	170	7242	7415	EV-A71	C1	100	Germany	2015	Stool: Human
9	KU641506	184	7156	7343	EV-A71	C1	100	Germany	2015	Stool: Human
10	MG367596	184	7152	7339	EV-A71	C1	100	Denmark	2016	Not Stated: Human
11	LR027532	0	7411	7414	EV-A71	C1	100	France	2016	Throat Swab: Human
12	MH484071	23	7368	7394	EV-A71	C1	100	Spain	2016	Not Stated: Human
13	MH484070	23	7368	7394	EV-A71	C1	100	Spain	2016	Not Stated: Human
14	LR027534	0	7412	7415	EV-A71	C1	100	France	2016	Mouth Swab: Human
15	MG367599	184	7152	7339	EV-A71	C1	100	Denmark	2016	Not Stated: Human
16	KU641508	184	7149	7336	EV-A71	C1	100	Germany	2015	Stool: Human
17	MH484067	23	7368	7394	EV-A71	C1	100	Spain	2016	Not Stated: Human
18	LR027522	0	7411	7414	EV-A71	C1	99	France	2016	Nasopharyngeal Aspirate: Human
19	MH484069	23	7368	7394	EV-A71	C1	100	Spain	2016	Not Stated: Human
20	MH484066	23	7368	7394	EV-A71	C1	100	Spain	2016	Not Stated: Human
21	LR027521	0	7411	7414	EV-A71	C1	100	France	2016	Throat Swab: Human
22	MN515037	23	7368	7394	EV-A71	C1	100	Spain	2016	Not Stated: Human
23	MH484068	23	7368	7394	EV-A71	C1	100	Spain	2016	Not Stated: Human
24	LR027546	0	7410	7414	EV-A71	C1	100	France	2016	Throat Swab: Human
25	LR027531	0	7409	7413	EV-A71	C1	100	France	2016	Nasopharyngeal Aspirate: Human
26	MG367598	184	7152	7339	EV-A71	C1	100	Denmark	2016	Not Stated: Human
27	LR027527	0	7411	7414	EV-A71	C1	100	France	2016	Throat Swab: Human
28	LR027524	0	7409	7413	EV-A71	C1	100	France	2016	Throat Swab: Human
29	LR027536	0	7411	7414	EV-A71	C1	100	France	2016	Stool: Human
30	LR027530	0	7411	7414	EV-A71	C1	100	France	2016	Stool: Human
31	LR027538	0	7411	7414	EV-A71	C1	100	France	2016	Stool: Human
32	LR027535	0	7411	7414	EV-A71	C1	100	France	2016	Stool: Human
33	LR027528	0	7411	7414	EV-A71	C1	100	France	2016	Nasopharyngeal Aspirate: Human
34	LR027537	0	7411	7414	EV-A71	C1	100	France	2016	Stool: Human
35	LC321990	89	7241	7334	EV-A71	C1	100	Japan	2017	Not Stated: Human
36	LC321991	76	7267	7345	EV-A71	C1	100	Japan	2017	Not Stated: Human
37	MT081373	0	7410	7413	EV-A71	C1	100	USA	2018	Not Stated: Human

38	LR027539	0	7413	7416	EV-A71	C1	100	France	2017	Throat Swab: Human
39	LR027525	0	7412	7416	EV-A71	C1	100	France	2016	Blood: Human
40	KY888026	193	7211	7407	EV-A71	C1	100	USA	2016	Stool: Human
41	MK800119	63	7335	7401	EV-A71	C1	100	USA	2018	Cerebrospinal Fluid: Human
42	MT081375	0	7408	7413	EV-A71	C1	100	USA	2018	Not Stated: Human
43	MH718269	561	6798	7362	EV-A71	C1	100	USA	2018	Throat Swab: Human
44	MT081374	0	7408	7413	EV-A71	C1	100	USA	2018	Not Stated: Human
45	MG367595	185	7142	7330	EV-A71	C1	100	Denmark	2014	Not Stated: Human
46	LR027533	0	7415	7416	EV-A71	C1	100	France	2016	Stool: Human
47	LR027529	-1	7412	7414	EV-A71	C1	100	France	2016	Throat Swab: Human
48	MG367594	184	7140	7330	EV-A71	C1	100	Denmark	2007	Not Stated: Human
49	LR027542	0	7410	7414	EV-A71	C1	100	Germany	2003	Not Stated: Human
50	KU641500	184	3149	3336	EV-A71	C1	100	Germany	2015	Stool: Human
51	KU641498	356	2977	3336	EV-A71	C1	100	Germany	2015	Stool: Human
52	KU641499	356	2977	3336	EV-A71	C1	100	Germany	2015	Stool: Human
53	DQ341361	0	7409	7413	EV-A71	C1	100	Australia	Not Stated	Not Stated: Human
54	AB747375	0	7409	7413	EV-A71	C1	100	Thailand	Not Stated	Not Stated: Not Stated
55	LR027544	0	7409	7413	EV-A71	C1	100	France	2003	Stool: Human
56	AB550340	0	7409	7413	EV-A71	C1	100	Malaysia	Not Stated	Not Stated: Not Stated
57	LR027541	618	6773	7390	EV-A71	C1	100	Netherlands	2005	Not Stated: Human
58	AB575937	90	7271	7363	EV-A71	C1	100	Netherlands	2001	Not Stated: Not Stated
59	AB550341	0	7409	7413	EV-A71	C1	100	Malaysia	Not Stated	Not Stated: Not Stated
60	DQ341358	0	7409	7413	EV-A71	C1	100	Malaysia	Not Stated	Not Stated: Not Stated
61	LT719067	0	7409	7413	EV-A71	C1	100	Cameroon	2008	Not Stated: Not Stated
62	DQ452074	0	7410	7413	EV-A71	C1	100	Norway	2003	Not Stated: Not Stated
63	DQ341360	0	7408	7413	EV-A71	C1	100	Malaysia	Not Stated	Not Stated: Not Stated
64	LR027543	0	7409	7413	EV-A71	C1	100	Germany	2008	Stool: Human
65	AB575938	26	7339	7370	EV-A71	C1	100	Netherlands	2010	Not Stated: Not Stated
66	EU414332	57	7254	7317	EV-A71	C1	100	Switzerland	Not Stated	Stool: Human
67	EU414335	51	7318	7373	EV-A71	C1	100	Switzerland	Not Stated	Plasma: Human
68	EU414334	51	7326	7381	EV-A71	C1	99	Switzerland	Not Stated	Plasma: Human
69	EU414333	51	7359	7414	EV-A71	C1	100	Switzerland	Not Stated	Cerebrospinal Fluid: Human
70	EU414331	51	7307	7364	EV-A71	C1	100	Switzerland	Not Stated	Lower respiratory tract: Human
71	MG367600	184	7140	7330	EV-A71	C1	98	Denmark	2016	Not Stated: Not Stated
72	JF738000	90	7329	7421	EV-A71	C1	100	Thailand	2009	Not Stated: Human
73	LR027547	0	7409	7413	EV-A71	C1	100	France	2006	Pharynx: Human
74	LR027545	0	7409	7413	EV-A71	C1	100	France	2007	Pharynx: Human
75	AB575936	61	7298	7365	EV-A71	C1	100	Netherlands	1991	Not Stated: Not Stated

76	MK652139	19	7389	7413	EV-A71	C1	100	USA	2018	Stool: Human
77	HQ647172	0	7421	7425	EV-A71	C1	100	Canada	1994	Not Stated: Not Stated
78	DQ341359	0	7409	7413	EV-A71	C1	99	Malaysia	Not Stated	Not Stated: Not Stated
79	AB575935	56	7305	7366	EV-A71	C1	100	Netherlands	1991	Not Stated: Not Stated
80	LC506514	0	7434	7438	EV-A71	C1	92	Japan	1990	Not Stated: Human
