# Supplementary figures and images for: Pan-Enterovirus Amplicon-Based High-Throughput Sequencing Detects the Complete Capsid of a EVA71 Genotype C1 Variant via Wastewater-Based Epidemiology in Arizona
Source: Viruses. 2021 Jan 7;13(1):74. doi: 10.3390/v13010074 (PMC7827028; doi:10.3390/v13010074)

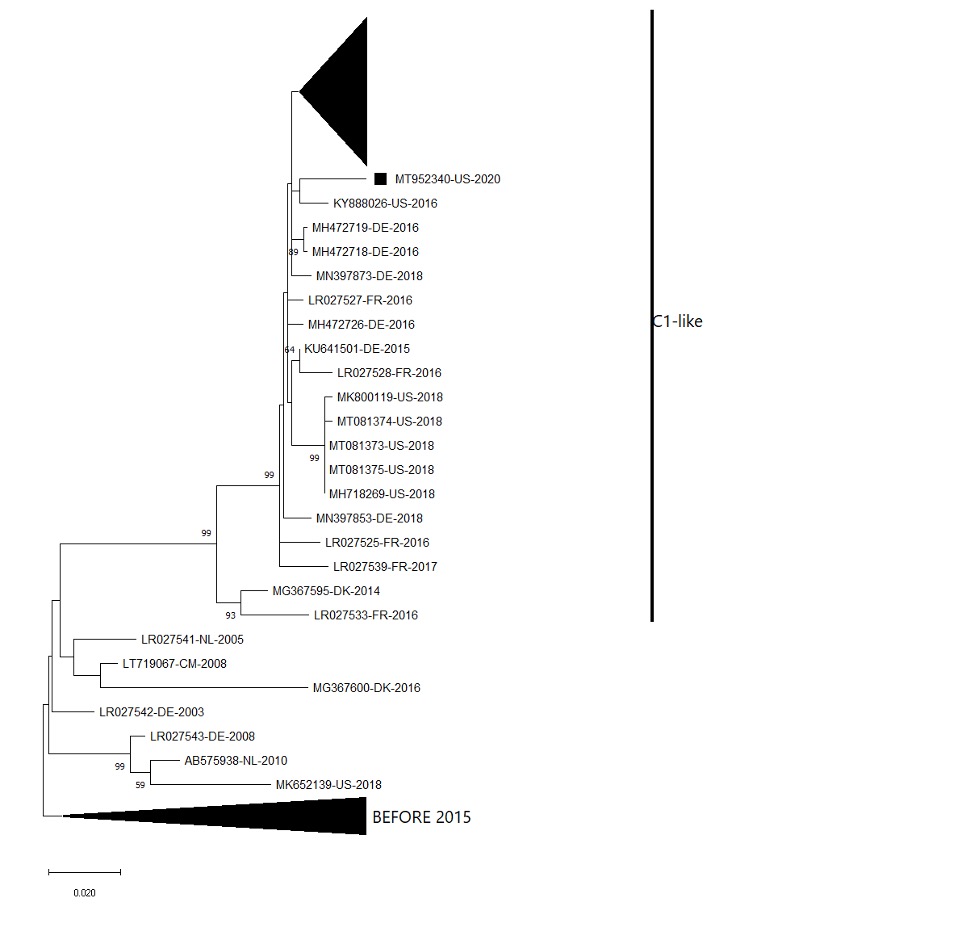

Supplement: Supplementary file 1 [file viruses-13-00074-s001.zip › figS1.jpg]

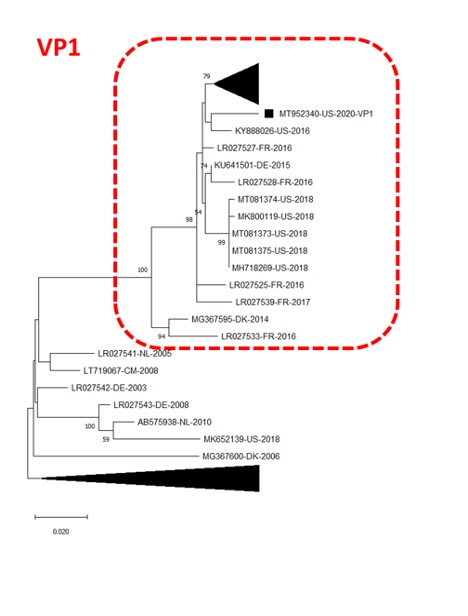

Supplement: Supplementary file 1 [file viruses-13-00074-s001.zip › figS2.jpg]

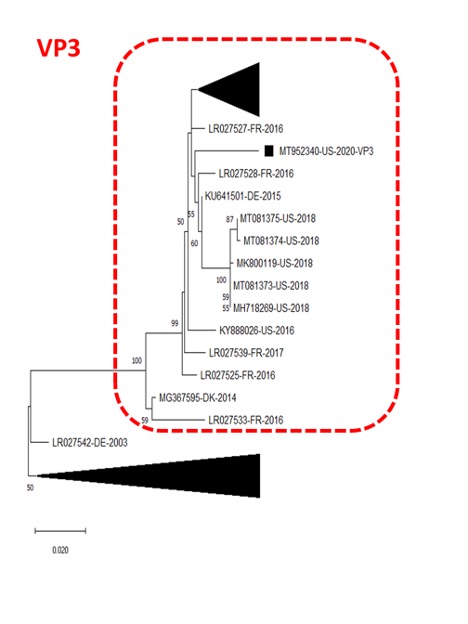

Supplement: Supplementary file 1 [file viruses-13-00074-s001.zip › figS3.jpg]

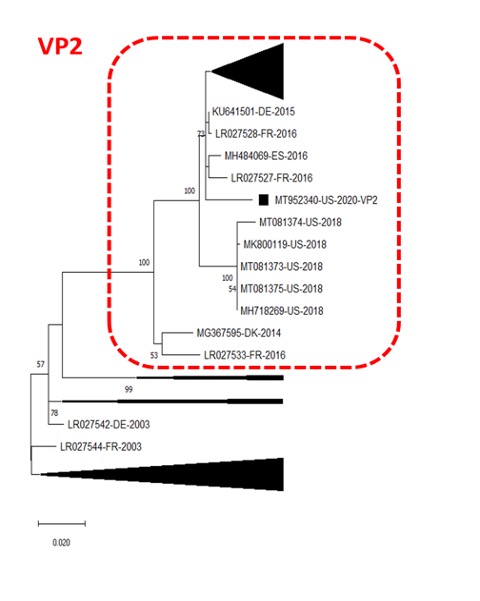

Supplement: Supplementary file 1 [file viruses-13-00074-s001.zip › figS4.jpg]

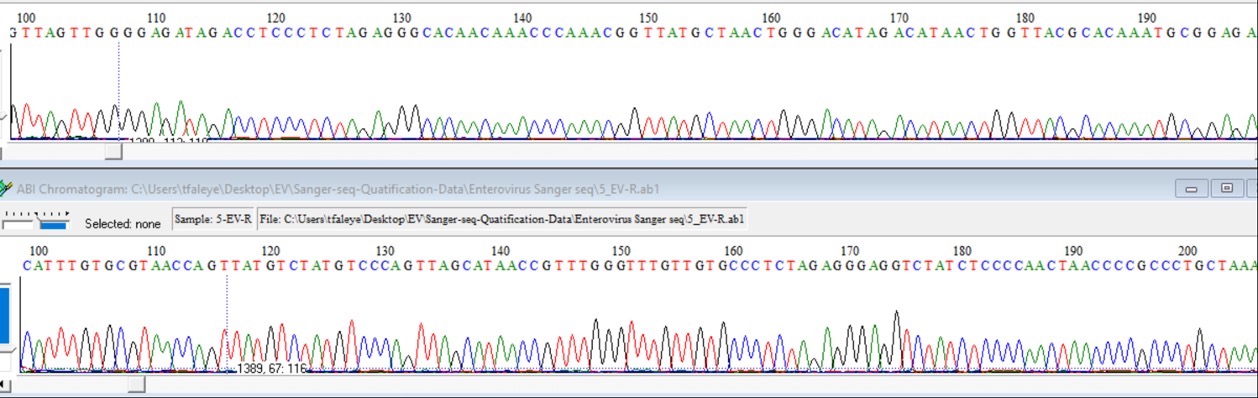

Supplement: Supplementary file 1 [file viruses-13-00074-s001.zip › figS5.jpg]

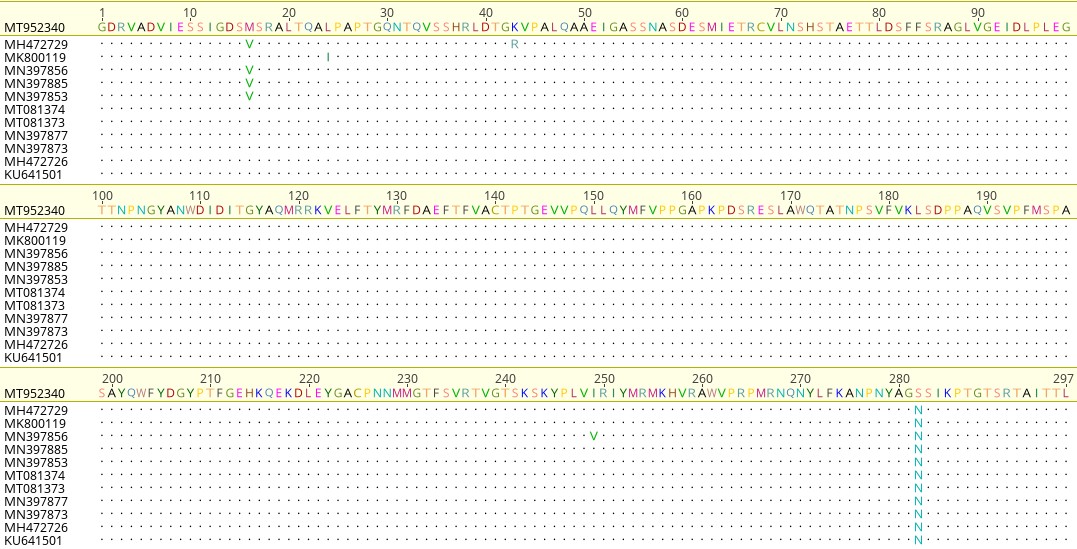

Supplement: Supplementary file 1 [file viruses-13-00074-s001.zip › figS6.jpg]
